# Additive Impact of Interleukin 6 and Neuron Specific Enolase for Prognosis in Patients With Out-of-Hospital Cardiac Arrest – Experience From the HAnnover COoling REgistry

**DOI:** 10.3389/fcvm.2022.899583

**Published:** 2022-05-31

**Authors:** Muharrem Akin, Jan-Thorben Sieweke, Vera Garcheva, Carolina Sanchez Martinez, John Adel, Pia Plank, Paris Zandian, Kurt-Wolfram Sühs, Johann Bauersachs, Andreas Schäfer

**Affiliations:** ^1^Cardiac Arrest Center, Department of Cardiology and Angiology, Hannover Medical School, Hanover, Germany; ^2^Department of Neurology, Hannover Medical School, Hanover, Germany

**Keywords:** out-of-hospital cardiac arrest, interleukin 6, neuron-specific enolase, neurological outcome, prognosis

## Abstract

**Background:**

Patients after out-of-hospital cardiac arrest (OHCA) are at increased risk for mortality and poor neurological outcome. We assessed the additive impact of interleukin 6 (IL-6) at admission to neuron-specific enolase (NSE) at day 3 for prognosis of 30-day mortality and long-term neurological outcome in OHCA patients.

**Methods:**

A total of 217 patients from the HAnnover COoling REgistry with return of spontaneous circulation (ROSC) after OHCA and IL-6 measurement immediately after admission during 2017–2020 were included to investigate the prognostic value and importance of IL-6 in addition to NSE obtained on day 3. Poor neurological outcome was defined by cerebral performance category (CPC) ≥ 3 after 6 months.

**Results:**

Patients with poor outcome showed higher IL-6 values (30-day mortality: 2,224 ± 524 ng/l vs 186 ± 15 ng/l, *p* < 0.001; CPC ≥ 3 at 6 months: 1,440 ± 331 ng/l vs 180 ± 24 ng/l, *p* < 0.001). IL-6 was an independent predictor of mortality (HR = 1.013/ng/l; 95% CI 1.007–1.019; *p* < 0.001) and poor neurological outcome (HR = 1.004/ng/l; 95% CI 1.001–1.007; *p* = 0.036). In ROC-analysis, AUC for IL-6 was 0.98 (95% CI 0.96–0.99) for mortality, but only 0.76 (95% CI 0.68–0.84) for poor neurological outcome. The determined cut-off value for IL-6 was 431 ng/l for mortality (NPV 89.2%). In patients with IL-6 > 431 ng/l, the combination with NSE < 46 μg/l optimally identified those individuals with potential for good neurological outcome (CPC ≤ 2).

**Conclusion:**

Elevated IL-6 levels at admission after ROSC were closely associated with 30-day mortality. The combination of IL-6 and NSE provided clinically important additive information for predict poor neurological outcome at 6 months.

## Introduction

High rates of mortality and poor neurological outcome limit short and long-term prognosis in patients with out-of-hospital cardiac arrest (OHCA) (1). Compromised circulation during cardiac arrest leads to cellular hypoxemia causing anaerobic metabolism and subsequent induction of cell death, a process which continuously progresses even after return of spontaneous circulation (ROSC) (2). This leads to an inflammatory sepsis-like syndrome, the so called post-cardiac arrest syndrome, with marked increase in cytokine levels as a sign of activated inflammation and hemostasis which in turn may lead to secondary brain injury and myocardial dysfunction (3). While interleukin 1 and TNF-α are the primary pro-inflammatory cytokines, interleukin 6 (IL-6) is considered as a major “messenger” cytokine (4).

IL-6 is associated with an increased immune response and an unfavorable outcome in asphyxic neonates (5). Besides its role as a differentiation factor for B-cells, IL-6 also regulates hematopoiesis, inflammation, and neurogenesis (6, 7). Elevated IL-6 levels also have been observed in cerebral ischemia (7). Cerebral ischemia results in release of several pro-inflammatory cytokines, including IL-6 (8). During cardiac arrest, blood-brain barrier permeability is increased leading to elevated serum interleukin concentrations (9, 10).

Increased IL-6 levels were associated with poor outcome in OHCA patients (11–14). While approaches with untargeted cytokine hemoadsorption after OHCA aiming for IL-6 reduction showed no benefit in survival (15, 16), the impact of targeted antibody therapy with an IL-6 receptor antagonist, tocilizumab, is currently investigated (17).

Here, we investigated the prognostic value of additive IL-6 measurements on top of routine sampling of neuron-specific enolase (NSE) to predict 30-day mortality and/or 6-month poor neurological outcome in patients from the HAnnover COoling REgistry (HACORE) population, since more recent analyses indicated that relying on NSE alone to predict poor neurological outcome with reasonable accuracy will require much higher cut-offs than previously recommended (18, 19). HACORE is suitable for this investigation since all OHCA patients receive standardized care according to a specific regimen (HaCRA) without routine application of early prognostication with intention of withdrawing life-support (20). Thereby, our cohort is not confounded by a self-fulfilling prophecy regarding poor outcome.

## Materials and Methods

### Study Design and Patients

HACORE, a prospective observational registry, is in accordance with the Declaration of Helsinki and approved by the ethics committee (#3567–2017) at Hannover Medical School. Written informed consent was obtained from legal guardians during the unconscious period and re-consented by survivors after gaining consciousness. HACORE includes anonymized data from all OHCA patients treated at our cardiac arrest center with a standardized protocol including therapeutic hypothermia (20).

In the present study, we included all adult patients (*n* = 217) with OHCA and ROSC at admission to our center between 2017 and 2020 including complete data for IL-6 values at hospital admission and follow-up data from HACORE (Supplementary Figure 1). Patients were followed up for the period of their hospital stay and data were extracted from the electronic hospital patient data management system. Discharge letters from rehabilitation facilities were collected. Telephone-follow up was performed to assess long-term data, and neurological outcome was assessed by the Glasgow-Pittsburgh cerebral performance category (CPC) after 6 months. CPC is recommended by the Utsetin guidelines and is used for outcome measure for post-arrest neurological deficits as result of hypoxic-ischemic insult and reperfusion injury (21). It consist of 5 categories defined as 1: good cerebral performance, 2: moderate cerebral disability, 3: severe cerebral disability, 4: coma or vegetative state and 5: brain death. For scientific assessment in generous it is divided in good (CPC 1 and 2) and poor (CPC 3-5) outcome (21).

Patients were excluded from the analysis when they had ongoing cardiopulmonary resuscitation at admission. Because IL-6 values are influenced by chronic alcohol abuse, OHCA-patients with history of chronic alcohol abuse were excluded (22).

### Patient Management After Out-of-Hospital Cardiac Arrest Using Hannover Cardiac Resuscitation Algorithm

The Hannover Cardiac Resuscitation Algorithm (HaCRA) for patients after OHCA was performed as described previously (20). In summary, this approach includes a standardized procedure for the care of patients with OHCA. Patients are screened in an interdisciplinary manner in the emergency room, stabilized and cared for to the extent to transfer the patients for further clarification by cardiac catheterization and computed tomographic examination. Subsequently, standardized care is provided in the cardiac ICU of the cardiac arrest center, where patients receive therapeutic hypothermia using an intravascular cooling catheter (Coolguard Quattro^®^, ZOLL Medical, San Jose, CA, United States) with the aim of maintaining a core body temperature of 32°C for 24 to 48 h, followed by a controlled rewarming phase of 0.25°C per hour (23). During intensive care, neurological monitoring included 24-h recording of a 3-pole EEG, regular assessment of pupils by an automated measuring device, and determination of markers by laboratory chemistry were performed. First blood samples were collected in the emergency room prior to any treatment. Out of this sample using standard serum tubes IL-6 was measured by immune assay (Roche, Electro-Chemi-Luminescent-Immuno Assay (ECLIA) test kit on Cobas 8000 auto-analyzer, Roche Diagnostics GmbH, Germany). Neuromarkers were determined from clinical routine blood samples taken at day 3 after admission using standard serum tubes by immune assay (for NSE even like IL-6 with ECLIA test kit and for S-100b with sandwich-immunoassay both determined on a Cobas 8000 auto-analyzer (Roche Diagnostics GmbH, Germany) (18). None of these components served at any time solely for decision making for neurological outcome nor for withdrawal of life support. Those were only considered as indicators to trigger further diagnostics, e.g., repetitive brain imaging to detect extensive anoxic brain injury. Nevertheless, despite rather weak level of evidence for prognostication its use has been recommended in different ways by certain guidelines (24–26). After acute intensive care and stabilization, patients were transferred to appropriate rehabilitation centers with neurological-intensive care to achieve an optimal neurological outcome.

### Statistical Analysis

Baseline characteristics are presented as frequencies (n) and percentages (%), means ± standard deviation (SD) for normally distributed variables, or median and interquartile ranges (IQR) for non-normally distributed variables. Normally distributed variables were compared by Student’s *t*-test and Mann–Whitney test for non-parametric data, respectively. All group comparisons of continuous measures were performed using Wilcoxon’s test, whereas the chi-square or Fisher’s exact test was used to assess categorical data. Univariate Cox regression was performed including all variables potentially associated with 30-day mortality and 6 month poor neurological outcome, respectively. Predictors were specified using a multivariate Cox regression analysis with those variables, which were statistically significant in univariate Cox regression analysis. Results from the regression analyses were displayed as hazard ratios (HRs) with 95% confidence intervals (CIs). Prior multi-collinearity was assessed by variance inflation factor (VIF). We assessed the predictive accuracy of IL-6 and NSE for 30-day survival and neurological outcome in the first 6 months according to CPC-score by receiver operating characteristic (ROC) curves. Results were expressed in terms of area under the curve (AUC) and 95% CI for this area. Cut-off values for prediction were defined as the cut-off point having the highest Youden index (Yi = sensitivity + specificity -1). Sensitivity, specificity, positive and negative predictive value, accuracy for determined cut-offs were calculated.

Statistical analyses were performed using SPSS Statistics 26 (IBM SPSS Statistics 26) and GraphPad Prism 6.0 (GraphPad Software, Inc., La Jolla, CA, United States) for creating figures. A two-sided *p*-value of < 0.05 was considered statistically significant.

## Results

### Patient Characteristics

**FIGURE 1:**
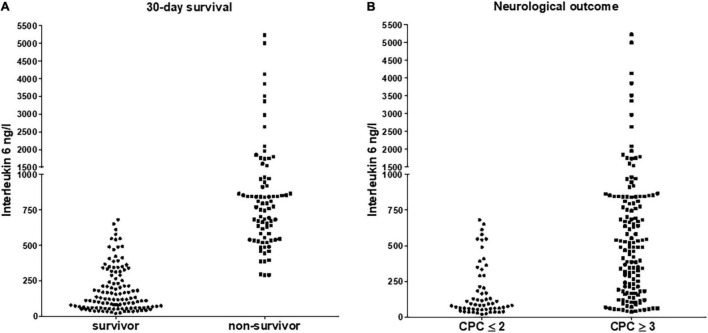
Distribution of interleukin 6 according to 30-day survival **(A)** for survivors and non-survivors and according to neurological outcome **(B)** for patients with good (CPC ≤ 2) and poor outcome (CPC ≥ 3), respectively. For both *p* was < 0.05.

Out of 217 patients identified and included for our analysis, 120 (55%) patients survived to day 30. Good neurological outcome at 6 months after OHCA defined by CPC of ≤ 2 was observed in 59 (27%) admitted patients (Table 1). Mean age was 62 ± 15 years, and younger age was more common in survivors (58 ± 16 vs. 67 ± 12 years, *p* < 0.001) as well as in patients with good neurological outcome (56 ± 14 vs. 65 ± 14 years, *p* < 0.001). A shockable first rhythm after ROSC was more common in patients surviving 30-days and those with good neurologic outcome. IL-6, NSE, S-100b and lactate were significantly higher in patients with poor neurological outcome and in patients dying within the first 30 days. Characteristics such as cardiovascular risk factors, history of atherosclerosis and manifestation of pre-existing health conditions were almost equally distributed between those groups (Table 1). Patients with poor outcome showed higher IL-6 values (30-day mortality: 2,224 ± 524 ng/l vs 186 ± 15 ng/l, *p* < 0.001; CPC ≥ 3 at 6 months: 1,440 ± 331 ng/l vs 180 ± 24 ng/l, *p* < 0.001). IL-6 in particular was a better discriminator regarding mortality than to detect good neurological outcome (Figure 1). On the other hand NSE and S-100b were more discriminative for neurological outcome than for mortality and lactate was unspecific (Supplementary Figure 2).

**TABLE 1:** Baseline characteristics and distribution according to 30-day survival and neurological outcome during first 6 month.

	All		30-day survival					Neurological outcome				
			Alive		Deceased			CPC ≤ 2		CPC ≥ 3		
	
	*n* = 217		*n* = 120		*n* = 97		*p* value	*n* = 59		*n* = 158		*p* value
Gender (male)	167	(77)	98	(82)	69	(71)	0.076	46	(78)	121	(77)	0.076
Age (years)	62 ± 15		58 ± 16		67 ± 12		< 0.001	56 ± 14		65 ± 14		< 0.001
Body mass index (kg/m^2^)	26.6	(24.6–29.4)	26.3	(24.4–29.3)	27.8	(26.1–31.0)	0.147	26.3	(24.3–29.4)	27.7	(24.6–29.6)	0.147
Arterial hypertension	120	(55)	66	(55)	54	(56)	1.000	30	(51)	90	(57)	1.000
Nicotine abuse	68	(31)	44	(37)	24	(25)	0.077	22	(37)	46	(29)	0.077
Diabetes	41	(19)	18	(15)	23	(24)	0.118	4	(7)	37	(23)	0.118
Hyperlipidemia	76	(35)	45	(38)	31	(32)	0.474	22	(37)	54	(34)	0.474
Positive family history for CAD	15	(7)	11	(9)	4	(4)	0.183	10	(17)	5	(3)	0.183
Heart failure	26	(12)	14	(12)	12	(12)	1.000	8	(14)	18	(11)	1.000
CHD	56	(26)	25	(21)	31	(32)	0.084	13	(22)	43	(27)	0.084
CABG	19	(9)	9	(8)	10	(10)	0.480	5	(8)	14	(9)	0.480
PCI	24	(11)	11	(9)	13	(13)	0.386	6	(10)	18	(11)	0.386
PAD	19	(9)	6	(5)	13	(13)	0.051	3	(5)	16	(10)	0.051
Stroke	23	(11)	12	(10)	11	(11)	0.829	2	(3)	21	(13)	0.829
CKI	21	(10)	8	(7)	13	(13)	0.110	4	(7)	17	(11)	0.110
RRT	3	(1)	0	(0)	3	(3)	0.088	0	(0)	3	(2)	0.088
Atrial fibrilation	30	(14)	16	(13)	14	(14)	0.845	7	(12)	23	(15)	0.845
COPD	33	(15)	12	(10)	21	(22)	0.022	2	(3)	31	(20)	0.022
Arrest setting							0.039					0.039
Home	124	(57)	60	(50)	64	(66)		23	(39)	101	(64)	0.076
Public	86	(40)	57	(48)	29	(30)		34	(58)	52	(33)	
Nursing home	4	(2)	1	(1)	3	(3)		1	(2)	3	(2)	0.147
Doctors office	0	(0)	0	(0)	0	(0)		0	(0)	0	(0)	1.000
Medical institution	3	(1)	2	(2)	1	(1)		1	(2)	2	(1)	
Working place	0	(0)	0	(0)	0	(0)		0	(0)	0	(0)	
Witnessed by lay	137	(63)	77	(64)	60	(62)	0.657	42	(71)	95	(60)	0.191
Witnessed by professional	37	(17)	24	(20)	13	(13)	0.423	8	(14)	29	(18)	1.000
Bystander CPR	162	(75)	94	(78)	68	(70)	0.209	50	(85)	112	(71)	0.037
Primary rhythm							< 0.001					0.001
Unknown	1	(0)	1	(1)	2	(2)		0	(0)	3	(2)	
Asystoly	70	(32)	22	(18)	48	(49)		8	(14)	62	(39)	
VF	115	(53)	84	(70)	31	(32)		49	(83)	66	(42)	
PEA	31	(14)	15	(13)	16	(16)		4	(7)	27	(17)	
Collapse to BLS/ALS (min)	2	(1–5)	0	(0–1)	0	(0–5)	0.012	0	(0–2)	0	(0–5)	0.008
BLS (min)	8	(5–10)	8	(5–10)	10	(5–10)	0.167	8	(5–10)	7	(5–10)	0.380
ALS (min)	15	(10–25)	11	(8–20)	20	(10–25)	0.006	10	(5–20)	17	(10–25)	0.040
ALS—BLS to ROSC (min)	20	(13–27)	16	(10–25)	25	(15–31)	0.003	15	(10–25)	20	(15–30)	0.043
ROSC (min)	20	(15–30)	15	(16–25)	25	(15–35)	< 0.001	15	(14–25)	20	(15–30)	0.012
Time collapse to EMS-arrival (min)	8.5	(5–10)	8	(5–10)	10	(5–10)	0.243	8	(5–10)	10	(5–10)	0.350
Catecholamines by EMS	173	(80)	84	(70)	89	(92)	< 0.001	41	(69)	132	(84)	0.036
IL-6 (ng/l)	1,097 ± 244		186 ± 15		2,224 ± 524		< 0.001	180 ± 24		1,440 ± 331		< 0.001
NSE (μg/l*)	85 ± 9		46 ± 6		191 ± 25		< 0.001	28 ± 3		115 ± 13		< 0.001
s-100b (μg/l*)	0.671 ± 0.233		0.488 ± 0.292		1.174 ± 0.330		0.193	0.104 ± 0.014		0.963 ± 0.349		0.016
Lactate (mmol/l)	7.82 ± 0.28		6.56 ± 0.36		9.36 ± 0.40		< 0.001	6.17 ± 0.52		8.42 ± 0.33		< 0.001
CK (U/l)	429 ± 51		397 ± 72		470 ± 71		0.477	536 ± 141		390 ± 46		0.327
CK-MB (U/l)	104 ± 7		92 ± 8		120 ± 12		0.054	101 ± 15		106 ± 8		0.762
hsTnT (ng/l)	626 ± 166		694 ± 273		541 ± 155		0.648	1,202 ± 558		416 ± 97		0.170
NT-proBNP (ng/l)	2,187 ± 365		1,442 ± 345		3,244 ± 715		0.026	1,263 ± 431		2,492 ± 461		0.054
CRP (mg/l)	10 ± 2		7 ± 1		15 ± 3		0.018	8 ± 2		11 ± 2		0.274
Procalcitonin (μg/l)	4.6 ± 0.6		2.7 ± 0.5		7.6 ± 1.3		< 0.001	3.0 ± 0.8		5.3 ± 0.8		0.079
Platelets (tsd/μl)	214 ± 5		212 ± 7		217 ± 8		0.672	208 ± 10		216 ± 6		0.469
Leukocytes (tsd/μl)	14.4 ± 0.4		14.2 ± 0.5		14.6 ± 0.7		0.577	14.3 ± 0.7		14.4 ± 0.5		0.962
Fibrinogen (g/l)	2.38 ± 0.13		2.46 ± 0.17		2.30 ± 0.22		0.554	2.27 ± 0.25		2.43 ± 0.16		0.594
Hospital stay (days)	10	(3–15)	15	(9–20)	4	(1–6)	0.018	16	(12–20)	8	(2–12)	0.024

*CABG, coronary artery bypass graft; CAD, chronic artery disease; CHD, chronic heart disease; CK, creatinine kinase; CKI, chronic kidney injury; COPD, chronic obstructive pulmonary disease; CPR, cardiopulmonary resuscitation; CRP, c reactive protein; EMS, emergency medical service; IL-6, interleukin 6; NSE, neuron specific enolase; PAD, peripheral artery disease; PEA, pulseless electric activity; PCI, percutaneous coronary intervention; ROSC, return of spontaneous circulation; RRT, renal replacement therapy; VF, ventricular fibrillation.*

*Measured at day 3 after admission.

### Univariate and Multivariate Cox Regression Analysis

Overall 30-day mortality was only associated with admission IL-6 levels (HR = 1.013/ng/l; 95% CI 1.007–1.019; *p* < 0.001) and NSE at day 3 after arrest (HR = 1.013/μg/l; 95% CI 1.004–1.022; *p* = 0.005) in multivariate analysis (Table 2A), whereas impaired neurological outcome including deceased patients was associated with IL-6 (HR = 1.004/ng/l; 95% CI 1.001–1.007; *p* = 0.036), NSE (HR = 1.018/μg/l; 95% CI 1.004–1.032; *p* = 0.011), a history of diabetes, non-public arrest and a primary non-shockable rhythm (Table 2B and Supplementary Figure 3).

**TABLE 2:** Univariate and Multivariate Cox regression Analysis for (a) 30-day survival and (b) poor neurological outcome.

	Univariate			Multivariate		
	HR	(95% CI)	*p* value	HR	(95% CI)	*p* value
**A) 30-Day Survival**						
IL-6 (ng/l)	1.012	(1.009–1.016)	< 0.001	1.013	(1.007–1.019)	< 0.001
NSE (μg/l)	1.015	(1.010–1.021)	< 0.001	1.013	(1.004–1.022)	0.005
Lactate (mmol/l)	1.189	(1.106–1.278)	< 0.001			ns
CRP (mg/l)	1.02	(1.003–1.038)	0.024			ns
Procalcitonin (μg/l)	1.1	(1.041–1.161)	0.001			ns
COPD	2.487	(1.154–5.358)	0.020			ns
ROSC (min)	1.05	(1.026–1.076)	< 0.001			ns
Shockable rhythm	0.201	(0.113–0.359)	< 0.001			ns
Catecholamines by EMS	4.768	(2.096–10.848)	< 0.001			ns
**B) Poor Neurological Outcome**						
IL-6 (ng/l)	1.004	(1.003–1.006)	< 0.001	1.004	(1.001–1.007)	0.036
NSE (μg/l)	1.027	(1.011–1.042)	0.001	1.018	(1.004–1.032)	0.011
Lactate (mmol/l)	1.153	(1.062–1.252)	0.001			ns
Diabetes	4.205	(1.428–12.377)	0.009	3.714	(1.003–13.749)	0.049
COPD	6.957	(1.610–30.065)	0.009			ns
Arrest setting	0.584	(0.376–0.908)	0.017	0.430	(0.216–0.856)	0.016
Bystander CPR	0.438	(0.199–0.964)	0.040			ns
ROSC (min)	1.035	(1.007–1.063)	0.014			ns
Shockable rhythm	0.146	(0.069–0.310)	< 0.001	0.287	(0.106–0.776)	0.014
Catecholamines by EMS	2.229	(1.112–4.469)	0.024			ns

*COPD, chronic obstructive pulmonary disease; CPR, cardiopulmonary resuscitation; CRP, c reactive protein; EMS, emergency medical service; IL-6, interleukin 6; NSE, neuron specific enolase; ROSC, return of spontaneous circulation.*

### Interquartile Proportions of IL-6 in Relation to 30-Day Survival

**FIGURE 2:**
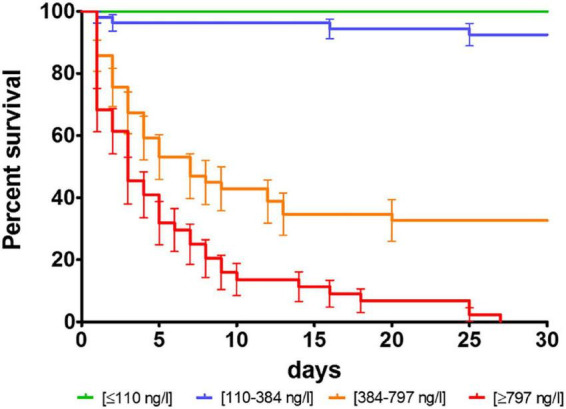
Survival proportions of interleukin 6 according to quartiles of HACORE population. *p* was < 0.0001 in log-rank test.

No patient within the lowest IL-6 quartile (<110 ng/l) of our population died within the first 30 days, whereas no patient within the highest quartile (>797 ng/l) survived longer than 27 days. Of patients in the second IL-6 quartile (110–384 ng/l) 9% and, of patients in the third IL-6 quartile (384–797 ng/l) 70% died within the first 30 days (Figure 2).

### Receiver Operating Characteristic Analysis and Definition of Cut-Off Values

**FIGURE 3:**
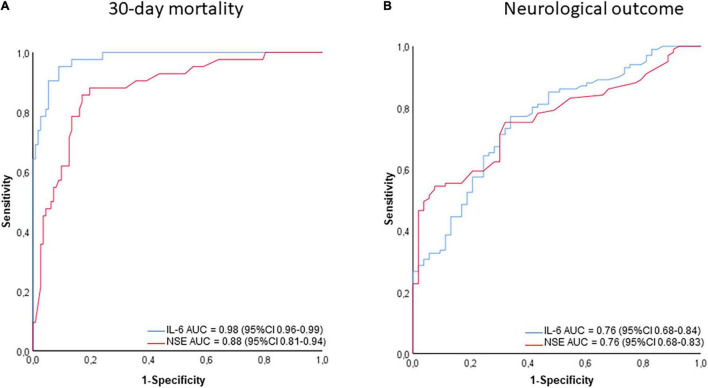
ROC curves for NSE and IL-6 for 30-day survival **(A)** and poor neurological outcome **(B)**. For both *p* was < 0.001.

The discriminative value of IL-6 and NSE to predict 30-day mortality and poor 6-months neurological outcome was determined using ROC analysis (Figure 3). The area under the curve was higher for both, IL-6 and NSE, predicting 30-day mortality than poor neurological outcome (AUC 0.98; 95% CI 0.96–0.99 vs. 0.76; 95% CI 0.68–0.84 for IL-6, and 0.88; 95% CI 0.81–0.94 vs. 0.76; 95% CI 0.68–0.083 for NSE, respectively). According to the highest Youden-Index a cut-off value for IL-6 of 431 ng/l (YI: 0.863) and for NSE of 46.5 μg/l (YI: 0.690) for 30-day mortality as well as a cut-off for IL-6 of 131 ng/l (YI: 0.433) and for NSE of 44.5 μg/l (YI: 0.469) for poor neurological outcome could be identified. Overall, IL-6 was most useful in predicting 30-day mortality while NSE was more helpful to discriminate long-term neurological outcome (Table 3). The lowest IL-6 value to predict good neurological outcome with a 100% specificity was 21 ng/l and highest value to predict poor outcome with a 100% specificity was 682 ng/l.

**TABLE 3:** Sensitivity, specificity and predictive values for identified cut-off values.

Endpoint	Marker	Cut-off	Sensitivity	(95% CI)	Specificity	(95% CI)	PPV	(95% CI)	NPV	(95% CI)
30-day survival	IL-6	431 ng/l	90.8	(84.2–95.3)	93.8	(87.0–97.7)	94.8	(89.3–97.5)	89.2	(82.5–93.7)
	NSE	46.5 μg/l	83.0	(74.8–89.5)	85.7	(71.5–94.6)	93.9	(88.1–97.2)	65.5	(55.2–74.4)
Poor neurological outcome	IL-6	131 ng/l	66.1	(52.6–77.9)	84.2	(77.5–89.5)	60.9	(51.1–70.0)	86.9	(82.2–90.5)
	NSE	44.5 μg/l	92.5	(81.8–97.9)	54.5	(44.2–64.4)	51.6	(45.9–57.2)	93.2	(84.1–97.3)

*CI, confidence interval; IL-6, interleukin 6; NPV, negative predictive value; NSE, neuron specific enolase; PPV, positive predictive value.*

### Survival Proportions and Prognostic Value According to Identified Cut-Off Values

Overall 47 patients died within the first 3 days. As NSE was originally determined only on day 3, there were no NSE values for these patients. For these 47 patients the median value of IL-6 at admission was 846 ng/l (IQR 643–1,876 ng/l). Regarding to the determined cut-off values for mortality in patients surviving for more than 3 days, 92 patients had lower values [IL-6 < 431 ng/l (median 103 ng/l; IQR 59–184 ng/l) and NSE < 46.5 μg/l (median 23 μg/l; IQR 18–30 μg/l)]. One of 92 (1%) died within the first 30 days. After 6 months, 48 out of 91 patients (53%) showed a good neurological outcome (CPC ≤ 2).

An IL-6 < 431 ng/l (median 213 ng/l; IQR 99–335 ng/l) and NSE > 46.5 μg/l (median 142 μg/l; IQR 86–178 μg/l) was present in 19 patients. Of those one patient (5%) died within the first 30 days. After 6 months, 3 out of 18 (17%) showed a good neurological outcome (CPC ≤ 2).

An IL-6 > 431 ng/l (median 612 ng/l; IQR 544–826) and NSE < 46.5 μg/l (median: 28 μg/l; IQR 22–35 μg/l) was present in 15 patients, while 5 out of 15 (33%) died within the first 30 days. After 6 months, 8 out of 15 (53%) showed a good neurological outcome (CPC ≤ 2) and 7 out of 15 (47%) showed a poor neurological outcome (CPC 3 or 4).

Values above the determined cut-off values [IL-6 > 431 ng/l (median: 804 ng/l; IQR 618–1,142 ng/l) and NSE > 46.5 μg/l (median: 189 μg/l; IQR 126–260 μg/l)] was present in 44 patients and 43 of them (98%) died within the first 30 days. After 6 months, the only one survivor had a poor neurological outcome (CPC = 4).

**FIGURE 4:**
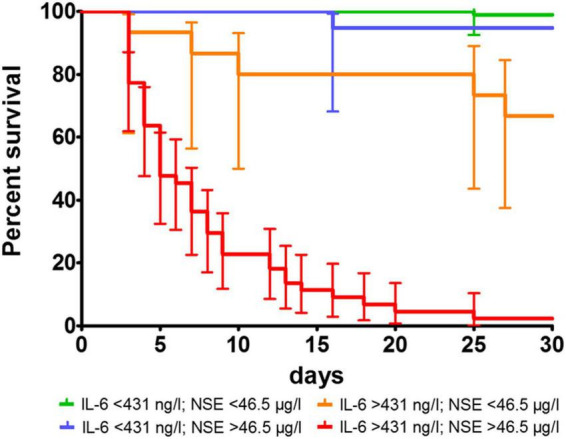
Survival proportions of combined determined cut-off values for mortality for IL-6 and NSE. *p* was < 0.0001 in log-rank test.

**FIGURE 5:**
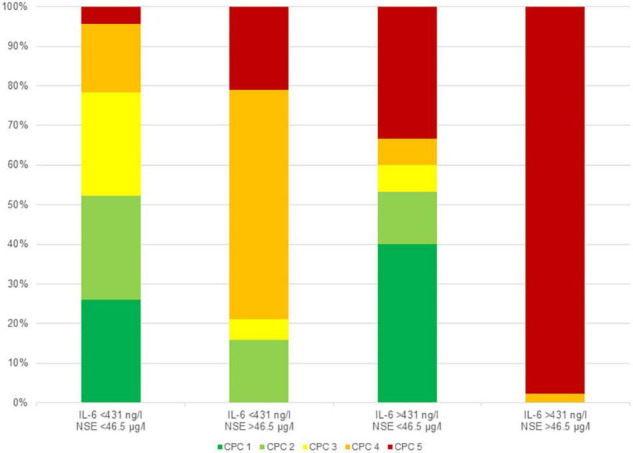
Neurological outcome according to CPC class in the corresponding groups depending to the determined cut-off values for mortality without patients dying during the first 3 days.

Overall, a value of IL-6 above the cut-off value has a more prognostic impact on the prediction of 30-day mortality than on neurological outcome. On the other hand, an elevated NSE value seems to be more predictive of poor neurological outcome than mortality. Exceeding both cut-offs is highly predictive for mortality (Figure 4). Importantly, low NSE values in patients with high IL-6 helps to identify patients who are likely to have a good neurological outcome if they survive the initial post-resuscitation phase (Figure 5).

## Discussion

Patients successfully resuscitated following OHCA and being treated within the HACORE cohort who died within 30 days or showed a less favorable neurological outcome at 6-months had higher levels of IL-6 immediately after hospital admission than survivors or those with good neurological outcome. While NSE was more favorable to predict poor neurological outcome, IL-6 was more strongly associated with 30-day overall survival. An IL-6 cut-off of 431 ng/l was best suited to predict 30-day mortality and a cut-off of 44.5 μg/l for NSE to predict neurological outcome.

Systemic ischemia/reperfusion response, cerebral injury and myocardial dysfunction as consequences of post-cardiac arrest syndrome are leading to a sepsis-like inflammatory reaction and release of interleukins in the brain as well as in the entire body (11, 27). IL-6 plays a key role as it is associated with leukocytes, vascular endothelium and parenchymal cells and is associated with unfavorable outcome even after cardiac arrest (17). Although IL-6 is present in the cerebrospinal fluid and brain, it remains undetermined whether the elevation in IL-6 levels after initiation of a systemic response by cardiac arrest is due to release from the cerebrospinal fluid or from necrotizing brain cells. For an exact differentiation, a direct sampling of cerebrospinal fluid would be necessary, but this is not feasible considering the urgent need for revascularization and antiplatelet therapy in many patients. Imaging using detection of previously labeled substances or cells could possibly help, but would be associated with a delay in the necessary primary care such as rapid induction of therapeutic hypothermia. Recognized effects of IL-6 include increased vascular permeability, fever, acute phase response, pro-coagulant effects, and induction of myocardial dysfunction (27). Also there are several observations in patients who have an increased release of IL-6 as a result of an infectious cause, which was accompanied by poor outcome (28). As a consequence, modulation of the systemic response has moved to the forefront as a therapeutic target to influence prognosis (29). Although an untargeted elimination and regulation of this response seems to be beneficial in systemic inflammatory response syndrome like in septic patients or in the setting of cardiac surgery, this may not be applicable for patients after cardiac arrest (15). However, there are new approaches that are more promising through targeted modulation (29, 30). The most recent approach is inhibiting IL-6 using tocilizumab, a monoclonal antibody against IL-6, in a post-cardiac arrest setting (17). The treatment with antibodies directed against IL-6 could reduce C-reactive protein and leukocyte levels as indicators for systemic inflammation as well as creatinine kinase myocardial band and troponin as indicators for myocardial injury (17). However, there was no benefit in survival or neurological outcome. Thus, the elimination of the inflammatory parameter, a potential factor leading to deterioration of neurological as well as cardiac function, will not be sufficient to cure these patients. Therefore, the goal remains in primary prevention of inflammation instead of secondary antagonism. One aspect might still be early initiation of therapeutic hypothermia in combination with causative treatment and high-quality intensive care medicine.

Neuroprognostication is a challenging task when treating post-arrest patients. While it is desirable to detect a futile outcome early during hospitalization, false-positive prognostication of poor outcome must be avoided. Current evidence regarding prognostication is weak (24). When using biomarkers for predicting neurological outcome, NSE is often used and recommended in guidelines, however, with differing cut-off levels (31, 32). While European guidelines use a cut-off above 60 ng/l for NSE as a marker of poor prognosis (32), we and others have recently shown that the false-positive prediction of poor neurological outcome by NSE at values between 60 and 100 ng/l is still too high (18, 19, 33). Trying to avoid the false-positive prediction when using NSE would result in a cut-off about 100 ng/l leading to many patients not being properly detected who will have a poor outcome. Although earlier studies have suggested to combine neuromarkers with other modalities trying to predict neurological prognosis, a NSE cut-off above 97 ng/ml had been suggested (34). Others have calculated different sometimes lower cut-offs, in particular when assessing the 72 h time-point (35). Depending on local protocols, cut-offs for one specific neuromarker might differ between populations. While the approach proposed by Daubin et al., suggesting the combination of NSE with non-biomarker predictors, is nowadays recommended by several guidelines, we believe that the combination of a neurological outcome-specific and a rather global mortality-related biomarker might further improve the quality and validity on the biomarker approach. Notwithstanding, the combined biomarker approach should still be accompanied by at least two other modalities (highly-malignant EEG, sever anoxic brain damage in cerebral imaging, non-reactive pupils in pupillometry, or bilateral absence of N20 peak in SSEP) in order to postulate a negative prognostication. Therefore, it might be of particular interest to combine certain easily measurable biomarkers, which assess different facets of the post-arrest syndrome. The combination of IL-6 and NSE appears to be a very promising approach allowing to combine lower thresholds for both parameters to predict a futile outcome. In particular, IL-6 helps to identify patients who are unlikely to survive and NSE further discriminates between likely good or poor neurological outcome. Therefore, we suggest to use both biomarkers in a sequential way.

### Limitations

This observational and retrospective registry-based analysis reflects a realistic picture of association of admission IL-6 with 30-day mortality and neurological outcome in patients with OHCA who underwent a standardized intrahospital care with early coronary diagnostics and, if necessary, intervention and hypothermia- according to the HaCRA protocol. However, there are some limitations to address. First of all data were extracted from a monocentric retrospective registry. It cannot be ruled out that there is a bias, since not all factors related to changes in IL-6 and NSE levels have been investigated. If IL-6 were to be used as a tool for early decision making in post-arrest care, such strategy needs to be tested in a randomized controlled trial. An additional cause of inconsistency between studies is the variability of techniques used to measure biomarkers, which can cause a significant systematic error between techniques. For these reasons, current guidelines do not recommend any particular biomarker threshold to predict poor outcome with 100% specificity. An additional caveat for use of biomarkers is represented by extracerebral sources, which may cause false positive results. For NSE these include red blood cells, neuroendocrine tumors, and small cell carcinoma.

## Conclusion

After out-of-hospital cardiac arrest IL-6 is elevated and particularly associated with 30-day mortality. Compared to established neuromarkers such as NSE, IL-6 seems to be less specific to predict neurological outcome. Therefore, it might be used as an early predictor for short-term mortality. Adjunctive use of NSE in patients identified as potential survivors by IL-6 can further discriminate between patients with favorable and unfavorable neurological outcome.

## Data Availability Statement

The original contributions presented in the study are included in the article/Supplementary Material, further inquiries can be directed to the corresponding author/s.

## Ethics Statement

The studies involving human participants were reviewed and approved by Hannover Medical School Ethics Committee (#3567–2017). The patients/participants provided their written informed consent to participate in this study.

## Author Contributions

MA and AS designed the registry, recruited patients, analyzed the data, and drafted the manuscript. J-TS, VG, CM, JA, PP, and PZ recruited patients, analyzed the data, and revised the manuscript. K-WS revised the manuscript. JB designed the registry and drafted manuscript. All authors contributed to the article and approved the submitted version.

## Conflict of Interest

The authors declare that the research was conducted in the absence of any commercial or financial relationships that could be construed as a potential conflict of interest.

## Publisher’s Note

All claims expressed in this article are solely those of the authors and do not necessarily represent those of their affiliated organizations, or those of the publisher, the editors and the reviewers. Any product that may be evaluated in this article, or claim that may be made by its manufacturer, is not guaranteed or endorsed by the publisher.

## Supplementary Material

Supplementary Figure 1 Flow chart of identified HACORE patients with OHCA in 2017–2020 and selection for further analyzed patient’s.

Supplementary Figure 2 Distribution of NSE **(A,D)**, S-100b **(B,E)** and lactate **(C,F)** according to 30-day survival **(A–C)** for survivors and non-survivors and according to neurological outcome **(D–F)** for patients with good (CPC ≤ 2) and poor outcome (CPC ≥ 3), respectively.

Supplementary Figure 3 Forest chart for univariate **(A,C)** and multivariate **(B,D)** analysis for 30-day survival **(A,B)** and poor neurological outcome **(C,D)**.

## Abbreviations

CPC: cerebral performance category
HACORE: HAnnover COoling REgistry
IL-6: interleukin 6
NSE: neuron-specific enolase
OHCA: out-of hospital cardiac arrest
ROSC: return of spontaneous circulation.
