# Supplementary figures and images for: Additive Impact of Interleukin 6 and Neuron Specific Enolase for Prognosis in Patients With Out-of-Hospital Cardiac Arrest – Experience From the HAnnover COoling REgistry
Source: Front Cardiovasc Med. 2022 May 31;9:899583. doi: 10.3389/fcvm.2022.899583 (PMC9194609; doi:10.3389/fcvm.2022.899583)

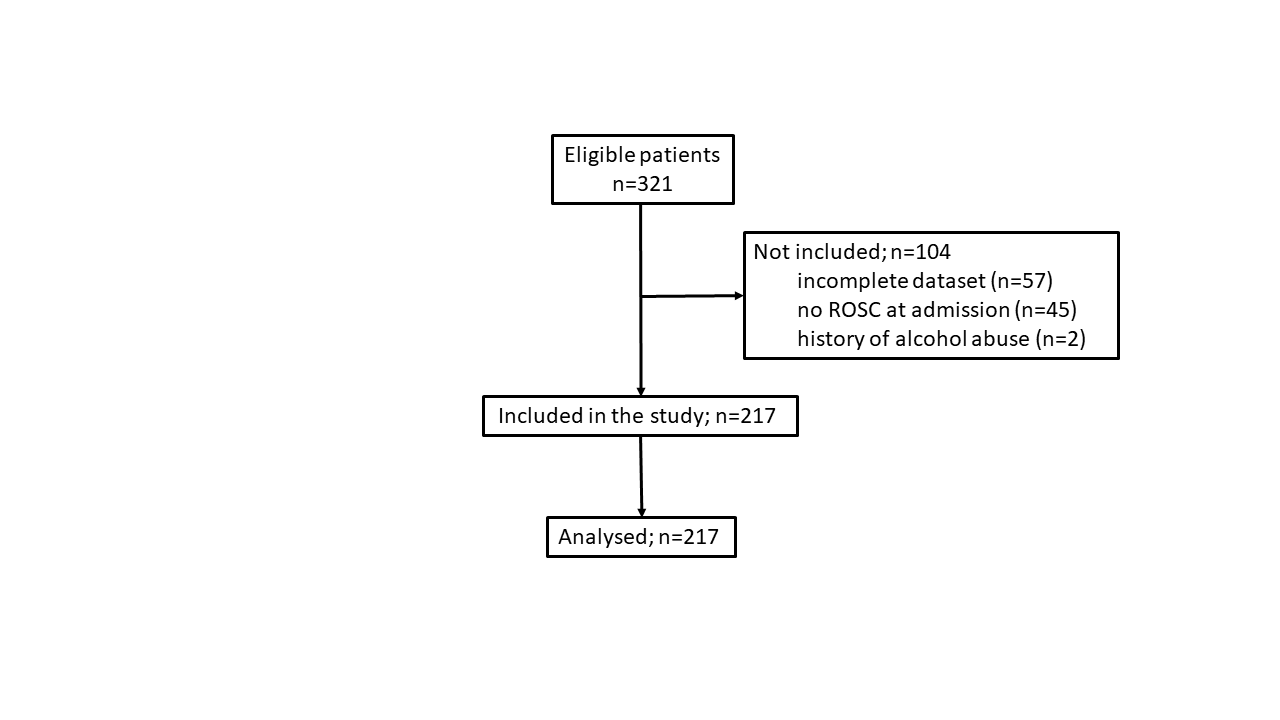

Supplement: Supplementary Figure 1 — Flow chart of identified HACORE patients with OHCA in 2017–2020 and selection for further analyzed patient’s. [file Image_1.tif]

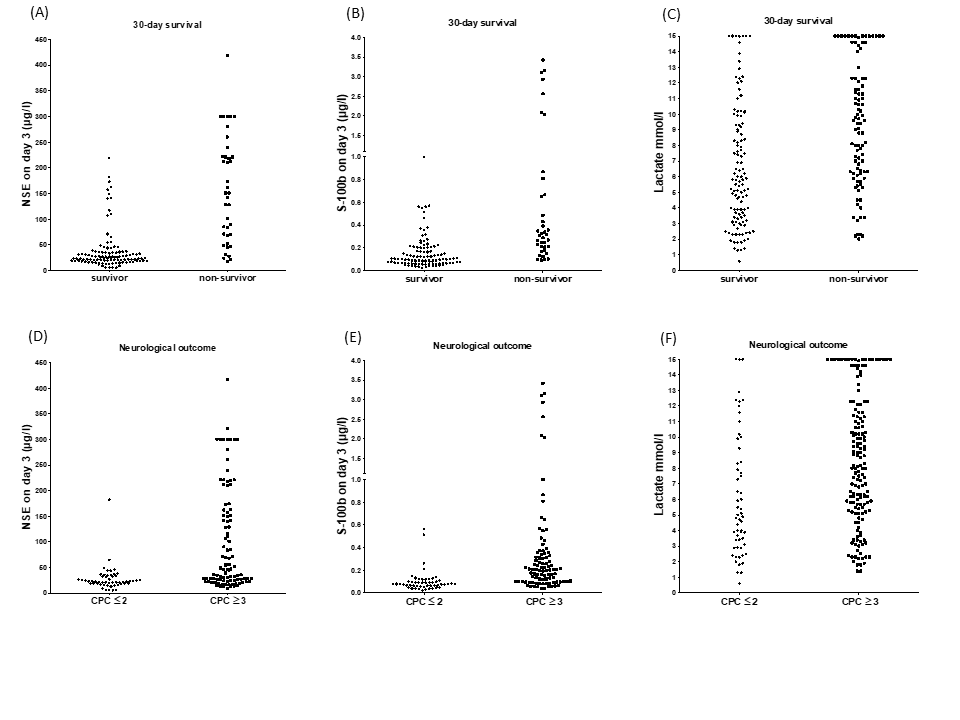

Supplement: Supplementary Figure 2 — Distribution of NSE (A,D), S-100b (B,E) and lactate (C,F) according to 30-day survival (A–C) for survivors and non-survivors and according to neurological outcome (D–F) for patients with good (CPC ≤ 2) and poor outcome (CPC ≥ 3), respectively. [file Image_2.tif]

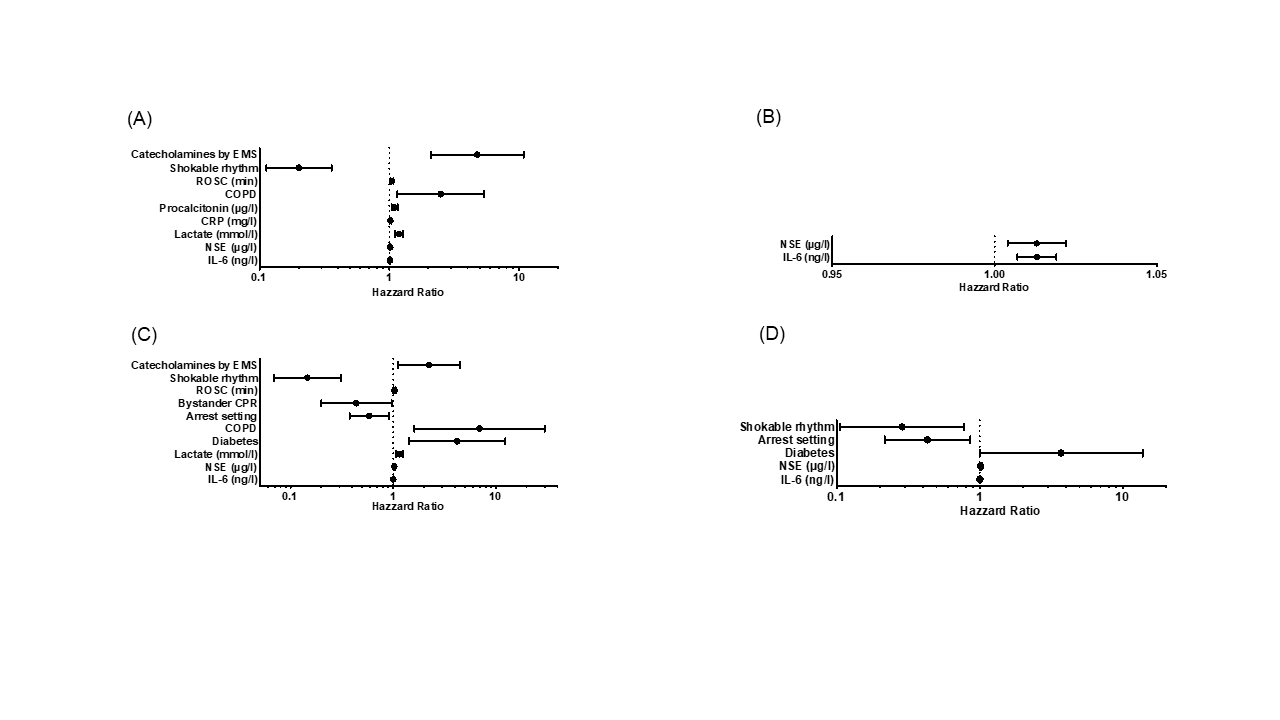

Supplement: Supplementary Figure 3 — Forest chart for univariate (A,C) and multivariate (B,D) analysis for 30-day survival (A,B) and poor neurological outcome (C,D). [file Image_3.tif]
